# Supplementary material for: Trait reactance and trust in doctors as predictors of vaccination behavior, vaccine attitudes, and use of complementary and alternative medicine in parents of young children
Source: PLoS One. 2020 Jul 27;15(7):e0236527. doi: 10.1371/journal.pone.0236527 (PMC7384640; doi:10.1371/journal.pone.0236527)
Supplement: S1 Table — (DOCX) [file pone.0236527.s001.docx]

**S1 Table.** Survey Questions Measuring Trust in Doctors and Vaccine Attitudes.

|  | **Survey question** | **Item label** |
| --- | --- | --- |
| **Trust** | I let doctors make the decisions concerning my health. | DocDecision |
|  | I feel heard when I visit the doctor. | DocHeard |
|  | I am satisfied with the medical treatment I receive from doctors. | DocSatisfied |
|  | I trust doctors' ability to make correct diagnoses. | DocDiagnose |
|  | When doctors make medical decisions, they have the patients’ best interest in mind. | DocPatientsBest |
|  | Doctors are too authoritative towards their patients. | DocAuthority^a^ |
| **Vaccine attitudes** | Vaccinating healthy children helps to protect others by stopping the spread of disease. | HerdImmunity |
|  | Children need vaccines for diseases that are not common anymore. | NotCommon |
|  | It is better to be immunized trough the disease than through the vaccine. | Immunized^a^ |
|  | Vaccines can cause autism. | Autism^a^ |
|  | Vaccines contain dangerous quantities of mercury. | Mercury^a^ |
|  | Childhood vaccines are safe. | ChildSafety |
|  | The risk of side effects outweighs the protective benefits of the childhood vaccines. | ChildSideEffects^a^ |
|  | Measles is a very serious disease. | ChildSerious |
|  | A good hygiene will make measles disappear from society – the vaccine is not necessary. | ChildNecessary^a^ |
|  | Childhood vaccines are effective in protecting against disease. | ChildProtection |
|  | The influenza vaccines are safe. | FluSafety |
|  | The risk of side effects outweighs the protective benefits of the influenza vaccines. | FluSideEffects^a^ |
|  | It is not worth getting the influenza vaccine, as the influenza symptoms are not serious. | FluSerious^a^ |
|  | Good hand hygiene and other preventive efforts are enough for avoiding the influenza even without vaccination. | FluNecessary^a^ |
|  | The influenza vaccines are effective in protecting against the disease. | FluProtection |

^a^Reverse-scored item.
