# Supplementary material for: Trait reactance and trust in doctors as predictors of vaccination behavior, vaccine attitudes, and use of complementary and alternative medicine in parents of young children
Source: PLoS One. 2020 Jul 27;15(7):e0236527. doi: 10.1371/journal.pone.0236527 (PMC7384640; doi:10.1371/journal.pone.0236527)
Supplement: S2 Table — (DOCX) [file pone.0236527.s002.docx]

**S2 Table.** Parents’ Responses to the Included Items of the HPRS.

|  | **Fully disagree** | |  | **Partly disagree** | |  | **Neither agree**  **nor disagree** | |  | **Partly agree** | |  | **Fully agree** | |
| --- | --- | --- | --- | --- | --- | --- | --- | --- | --- | --- | --- | --- | --- | --- |
| **Variable** | ***n*** | ***%*** |  | ***n*** | ***%*** |  | ***n*** | ***%*** |  | ***n*** | ***%*** |  | ***n*** | ***%*** |
| **Item 1** | 171 | 22.35 |  | 275 | 35.95 |  | 124 | 16.21 |  | 183 | 23.92 |  | 12 | 1.57 |
| **Item 2** | 257 | 33.73 |  | 242 | 31.76 |  | 102 | 13.39 |  | 142 | 18.64 |  | 19 | 2.49 |
| **Item 4** | 42 | 5.51 |  | 145 | 19.03 |  | 101 | 13.25 |  | 368 | 48.29 |  | 106 | 13.91 |
| **Item 6** | 93 | 12.20 |  | 271 | 35.56 |  | 130 | 17.06 |  | 245 | 32.15 |  | 23 | 3.02 |
| **Item 8** | 69 | 9.13 |  | 230 | 30.42 |  | 130 | 17.20 |  | 288 | 38.10 |  | 39 | 5.16 |
| **Item 9** | 421 | 55.18 |  | 253 | 33.16 |  | 50 | 6.55 |  | 35 | 4.59 |  | 4 | 0.52 |
| **Item 12** | 99 | 13.03 |  | 215 | 28.29 |  | 207 | 27.24 |  | 190 | 25.00 |  | 49 | 6.45 |
| **Item 13** | 211 | 27.84 |  | 277 | 36.54 |  | 150 | 19.79 |  | 105 | 13.85 |  | 15 | 1.98 |
| **Item 14** | 429 | 56.23 |  | 181 | 23.72 |  | 93 | 12.19 |  | 48 | 6.29 |  | 12 | 1.57 |
