# Supplementary material for: Trait reactance and trust in doctors as predictors of vaccination behavior, vaccine attitudes, and use of complementary and alternative medicine in parents of young children
Source: PLoS One. 2020 Jul 27;15(7):e0236527. doi: 10.1371/journal.pone.0236527 (PMC7384640; doi:10.1371/journal.pone.0236527)
Supplement: S3 Table — (DOCX) [file pone.0236527.s003.docx]

|  | **Fully disagree** | |  | **Partly disagree** | |  | **Partly agree** | |  | **Fully agree** | |
| --- | --- | --- | --- | --- | --- | --- | --- | --- | --- | --- | --- |
| **Statement label** | ***n*** | ***%*** |  | ***n*** | ***%*** |  | ***n*** | ***%*** |  | ***n*** | ***%*** |
| Trust | | | | | | | | | | | |
| **DocDecision** | 36 | 4.75 |  | 161 | 21.24 |  | 445 | 58.71 |  | 116 | 15.30 |
| **DocHeard** | 14 | 1.85 |  | 68 | 8.98 |  | 381 | 50.33 |  | 294 | 38.84 |
| **DocSatisfied** | 13 | 1.72 |  | 39 | 5.15 |  | 369 | 48.75 |  | 336 | 44.39 |
| **DocDiagnose** | 12 | 1.59 |  | 65 | 8.60 |  | 427 | 56.48 |  | 252 | 33.33 |
| **DocPatientsBest** | 11 | 1.45 |  | 48 | 6.34 |  | 314 | 41.48 |  | 384 | 50.73 |
| **DocAuthority^a^** | 156 | 20.74 |  | 384 | 51.06 |  | 194 | 25.80 |  | 18 | 2.39 |
| **VaccAttChild** | | | | | | | | | | | |
| **HerdImmunity** | 6 | 0.79 |  | 7 | 0.92 |  | 67 | 8.83 |  | 679 | 89.64 |
| **NotCommon** | 7 | 0.93 |  | 34 | 4.50 |  | 200 | 26.49 |  | 514 | 68.08 |
| **Immunized^a^** | 406 | 53.56 |  | 254 | 33.51 |  | 90 | 11.87 |  | 8 | 1.06 |
| **Autism^a^** | 422 | 56.57 |  | 241 | 32.31 |  | 72 | 9.65 |  | 11 | 1.47 |
| **Mercury^a^** | 491 | 65.73 |  | 204 | 27.31 |  | 39 | 5.22 |  | 13 | 1.74 |
| **ChildSafety** | 10 | 1.32 |  | 35 | 4.64 |  | 255 | 33.77 |  | 455 | 60.26 |
| **ChildSideEffects^a^** | 489 | 64.51 |  | 195 | 25.73 |  | 45 | 5.94 |  | 29 | 3.83 |
| **ChildSerious** | 11 | 1.46 |  | 41 | 5.45 |  | 212 | 28.19 |  | 488 | 64.89 |
| **ChildNecessary^a^** | 607 | 80.18 |  | 122 | 16.12 |  | 17 | 2.25 |  | 11 | 1.45 |
| **ChildProtection** | 3 | 0.40 |  | 20 | 2.64 |  | 137 | 18.10 |  | 597 | 78.86 |
| **VaccAttFlu** | | | | | | | | | | | |
| **FluSafety** | 26 | 3.44 |  | 118 | 15.61 |  | 327 | 43.25 |  | 285 | 37.70 |
| **FluSideEffects^a^** | 310 | 41.28 |  | 234 | 31.16 |  | 154 | 20.51 |  | 53 | 7.06 |
| **FluSerious^a^** | 316 | 41.91 |  | 293 | 38.86 |  | 123 | 16.31 |  | 22 | 2.92 |
| **FluNecessary^a^** | 181 | 23.91 |  | 282 | 37.25 |  | 255 | 33.69 |  | 39 | 5.15 |
| **FluProtection** | 35 | 4.62 |  | 206 | 27.21 |  | 412 | 54.43 |  | 104 | 13.74 |

**S3 Table.** Parent’s Responses to Statements Measuring Trust in Doctors and Attitudes to Vaccines.

The statements that the labels represent are described in S1 Table in the manuscript. ^a^Reverse-scored statement
