# Supplementary material for: Trait reactance and trust in doctors as predictors of vaccination behavior, vaccine attitudes, and use of complementary and alternative medicine in parents of young children
Source: PLoS One. 2020 Jul 27;15(7):e0236527. doi: 10.1371/journal.pone.0236527 (PMC7384640; doi:10.1371/journal.pone.0236527)
Supplement: S4 Table — (DOCX) [file pone.0236527.s004.docx]

**S4 Table.** Factor Loadings and Variances from Confirmatory Factor Analyses

|  |  | **Unstandardized** | |  | **Standardized** | | |
| --- | --- | --- | --- | --- | --- | --- | --- |
| **Factor** | **Parameter** | **Estimate** | ***SE*** |  | **Estimate** | ***SE*** | ***R*^2^** |
| **Factor loadings** | | | | | | | |
| **Reactance** | **Item 1** | 1.00 | − |  | 0.63 | 0.03 | .40 |
|  | **Item 2** | 0.79 | 0.06 |  | 0.50 | 0.03 | .25 |
|  | **Item 4** | 0.45 | 0.06 |  | 0.28 | 0.04 | .08 |
|  | **Item 6** | 1.00 | 0.06 |  | 0.63 | 0.03 | .40 |
|  | **Item 8** | 0.96 | 0.06 |  | 0.61 | 0.03 | .37 |
|  | **Item 9** | 1.14 | 0.07 |  | 0.72 | 0.03 | .52 |
|  | **Item 12** | 0.87 | 0.06 |  | 0.55 | 0.03 | .31 |
|  | **Item 13** | 1.05 | 0.06 |  | 0.66 | 0.03 | .44 |
|  | **Item 14** | 1.01 | 0.06 |  | 0.64 | 0.03 | .41 |
| **Trust** | **DocDecision** | 1.00 | − |  | 0.38 | 0.04 | .18 |
|  | **DocHeard** | 1.89 | 0.18 |  | 0.72 | 0.03 | .54 |
|  | **DocSatisfied** | 2.13 | 0.21 |  | 0.81 | 0.02 | .60 |
|  | **DocDiagnose** | 2.34 | 0.23 |  | 0.90 | 0.02 | .71 |
|  | **DocPatientsBest** | 2.10 | 0.20 |  | 0.80 | 0.02 | .70 |
|  | **DocAuthority** | 1.29 | 0.15 |  | 0.49 | 0.03 | .31 |
| **VaccAttGeneral** | **HerdImmunity** | 1.00 | − |  | 0.78 | 0.05 | .61 |
|  | **NotCommon** | 0.88 | 0.06 |  | 0.69 | 0.03 | .47 |
|  | **Immunized** | 0.92 | 0.05 |  | 0.72 | 0.03 | .51 |
|  | **Autism** | 0.62 | 0.06 |  | 0.49 | 0.04 | .24 |
|  | **Mercury** | 0.83 | 0.05 |  | 0.65 | 0.03 | .43 |
|  | **ChildSafety** | 0.99 | 0.06 |  | 0.78 | 0.02 | .60 |
|  | **ChildSideEffects** | 0.81 | 0.06 |  | 0.63 | 0.03 | .40 |
|  | **ChildSerious** | 0.68 | 0.06 |  | 0.54 | 0.04 | .29 |
|  | **ChildNecessary** | 0.94 | 0.06 |  | 0.73 | 0.04 | .54 |
|  | **ChildProtection** | 1.04 | 0.06 |  | 0.82 | 0.03 | .66 |
| **VaccAttFlu** | **FluSafety** | 1.00 | − |  | 0.87 | 0.02 | .75 |
|  | **FluSideEffects** | 1.00 | 0.03 |  | 0.86 | 0.02 | .74 |
|  | **FluSerious** | 0.78 | 0.03 |  | 0.67 | 0.03 | .45 |
|  | **FluNecessary** | 0.70 | 0.03 |  | 0.60 | 0.03 | .36 |
|  | **FluProtection** | 0.82 | 0.03 |  | 0.71 | 0.02 | .50 |
| **Factor variances** | | | | | | | |
| **Reactance** |  | 0.40 | 0.04 |  | 1.00 | − | − |
| **Trust** |  | 0.15 | 0.03 |  | 1.00 | − | − |
| **VaccAttGeneral** |  | 0.61 | 0.07 |  | 1.00 | − | − |
| **VaccAttFlu** |  | 0.75 | 0.03 |  | 1.00 | − | − |

Residual correlations between Item 4 and Item 6 (*r* = .39, *SE* = .04, *t* = 10.47, *p* < .001), DocHeard and DocSatisfied (*r* = .51, *SE* = .05, *t* = 10.94, *p* < .001), Autism and Mercury (*r* = .31, *SE* = .05, *t* = 6.05, *p* < .001), and FluSerious and FluNecessary (*r* = .31, *SE* = .04, *t* = 8.21, *p* < .001).
