# Supplementary material for: Trait reactance and trust in doctors as predictors of vaccination behavior, vaccine attitudes, and use of complementary and alternative medicine in parents of young children
Source: PLoS One. 2020 Jul 27;15(7):e0236527. doi: 10.1371/journal.pone.0236527 (PMC7384640; doi:10.1371/journal.pone.0236527)
Supplement: S1 Preregistration — (DOCX) [file pone.0236527.s005.docx]

**S1 Pre-registration.** Transparent Changes.^1^

1. Description of change: *Additional exclusion of 16 items from the CAM use measure*
   1. Rationale: *We applied an additional criterion for the inclusion of CAM items. The items had to not be included in the Finnish Current Care Guidelines (national evidence-based guidelines for the treatment and prevention of diseases in medical practice). This criterion was applied to get a definition of CAM that corresponds to the research question, that is, health behavior that goes against conventional medicine.*
   2. Effect of change on study results: *Results give information only about the use of such CAM treatments and substances that are not considered evidence-based.*
2. Description of change: *We have split the planned SR model into two models. One with vaccine attitudes as outcome measures, and another with vaccination behavior and CAM use as outcome measures.*
   1. Rationale: *Interpretation of results simplified.*
   2. Effect of change on study results: *None expected. Associations relevant for the hypotheses are represented in the two models.*
3. Description of change: *The vaccine attitudes factor was split into two factors*
   1. Rationale: *CFA indicated a two-factor structure (described in more detail in the manuscript)*
   2. Effect of change on study results: *Results relating to vaccine attitudes are obtained for the two vaccine categories separately.*
4. Description of change: *Sample size 770 instead of 771.*
   1. Rationale: *One individual had been included twice in the original data file.*
   2. Effect of change on study results: *None expected.*
5. Template by Mellor D, Esposito J, Hardwicke TE, Nosek BA, Cohoon J, Soderberg CK, ... Speidel R. Preregistration Challenge: Plan, Test, Discover. <https://osf.io/x5w7h/> (accessed 5 June 2020).
