# Supplementary material for: Trait reactance and trust in doctors as predictors of vaccination behavior, vaccine attitudes, and use of complementary and alternative medicine in parents of young children
Source: PLoS One. 2020 Jul 27;15(7):e0236527. doi: 10.1371/journal.pone.0236527 (PMC7384640; doi:10.1371/journal.pone.0236527)
Supplement: S1 Questionnaire — (DOCX) [file pone.0236527.s006.docx]

**S1 Questionnaire: Questionnaire translated into English**

Doctors

Please read the statements below and indicate to which degree you agree or disagree to each statement, by selecting a number on the scale from 1 (*Strongly disagree*) to 4 (*Strongly agree*).

|  | Strongly disagree |  |  | Strongly agree |
| --- | --- | --- | --- | --- |
| I let doctors make the decisions concerning my health | 1 | 2 | 3 | 4 |
| I feel heard when I visit the doctor | 1 | 2 | 3 | 4 |
| I am satisfied with the medical treatment I receive from doctors | 1 | 2 | 3 | 4 |
| I trust doctors’ ability to make correct diagnoses | 1 | 2 | 3 | 4 |
| When doctors make medical decisions, they have the patients’ best interest in mind | 1 | 2 | 3 | 4 |
| Doctors are too authoritative towards their patients | 1 | 2 | 3 | 4 |

Other treatments

Indicate by ticking the relevant boxes below, which of the following preparations or treatments you have used during the past 12 months, for the purpose of maintaining good health or treat an illness.

□ Vitamins and minerals (e.g., A, B, C, D, E, fluorine, iodine, zinc, potassium, calcium, magnesium, sodium)
□ Probiotics (e.g., lactic acid bacterium)
□ Fish oil and fatty acids (e.g., omega)
□ Colloidal silver
□ Turmeric
□ Ginger preparations
□ Health powders (e.g., maca, matcha and chlorella)
□ Natural products to treat flu

□ Aloe vera
□ Kombucha
□ Raw food

□ Vegetarian or vegan diet
□ Gluten-free diet
□ Lactose-free diet
□ LCHF-diet (low carb, high fat diet)
□ 5:2 diet
□ Mediterranean diet

□ Paleo diet

□ Fasting
□ Mindfulness
□ Meditation
□ Yoga
□ Tai chi
□ Chiropractic
□ Acupuncture

□ Cupping
□ Healing (e.g., distant healing, color healing, sound healing, crystal healing, healing minerals)

□ Prayer and laying on of hands

□ Energy treatment
□ Reiki

□ Rosen method
□ Zone therapy
□ Salt therapy

□ Chakra therapy

□ Homeopathy
□ Traditional Chinese medicine
□ Oil-pulling

□ Ayurveda
□ Detox

Vaccines

*Childhood vaccines* refer to the vaccines included in the vaccination program in Finland for children six years old and younger. The vaccination program includes the following vaccines: the rotavirus vaccine, pneumococcal conjugate vaccine (PCV; against meningitis, pneumonia, sepsis, and ear infection), DTaP-IPV-Hib (”5-in-1”) vaccine (against diphtheria, tetanus, whooping cough, polio, and Hib-diseases such as meningitis, epiglottitis, and sepsis), MMR vaccine (against measles, mumps, and rubella), DtaP-IPV (”4-in-1”) vaccine (against diphtheria, tetanus, whooping cough, and polio), and the chicken-pox vaccine.

*Influenza vaccines* refer to the seasonal vaccines for influenza.

Please read the statements below and indicate how much you agree with the statements by selecting a number between 1 (*Strongly disagree*) to 4 (*Strongly agree*).

|  | Strongly disagree |  |  | Strongly agree |
| --- | --- | --- | --- | --- |
| Vaccinating healthy children helps to protect others by stopping the spread of disease | 1 | 2 | 3 | 4 |
| Measles is a very serious disease | 1 | 2 | 3 | 4 |
| It is better to be immunized trough the disease than through vaccines | 1 | 2 | 3 | 4 |
| It is not worth getting the influenza vaccine as the influenza symptoms are not serious | 1 | 2 | 3 | 4 |
| Vaccines can cause autism | 1 | 2 | 3 | 4 |
| A good hygiene will make measles disappear from society – the vaccine is not necessary | 1 | 2 | 3 | 4 |
| Childhood vaccines are safe | 1 | 2 | 3 | 4 |
| The influenza vaccines are safe | 1 | 2 | 3 | 4 |
| The risk of side effects outweighs the protective benefits of childhood vaccines | 1 | 2 | 3 | 4 |
| The risk of side effects outweighs the protective benefits of influenza vaccines | 1 | 2 | 3 | 4 |
| Vaccines contain dangerous quantities of mercury | 1 | 2 | 3 | 4 |
| Children need vaccines for diseases that are not common anymore | 1 | 2 | 3 | 4 |
| Good hand hygiene and other preventive efforts are enough for avoiding the influenza even without vaccination | 1 | 2 | 3 | 4 |
| Childhood vaccines are effective in protecting against disease | 1 | 2 | 3 | 4 |
| The influenza vaccines are effective in protecting against the disease | 1 | 2 | 3 | 4 |

Have you ever hesitated in letting your child(ren) receive any of the childhood vaccines?

1. No

2. Yes

Have you ever postponed a childhood vaccination for your child(ren)?

1. No

2. Yes

Have you ever decided not to let your child(ren) receive any of the childhood vaccines?

1. No

2. Yes

Did you take the last influenza vaccine (season 2017-2018)?

1. No

2. Yes
