# Supplementary material for: Trait reactance and trust in doctors as predictors of vaccination behavior, vaccine attitudes, and use of complementary and alternative medicine in parents of young children
Source: PLoS One. 2020 Jul 27;15(7):e0236527. doi: 10.1371/journal.pone.0236527 (PMC7384640; doi:10.1371/journal.pone.0236527)
Supplement: S2 Questionnaire — (DOCX) [file pone.0236527.s007.docx]

**S2 Questionnaire: Questionnaire in Swedish**

Läkare

Vänligen läs påståendena nedan och ange för varje påstående hur mycket du håller med, genom att ringa in en siffra på skalan 1 (*Helt av annan åsikt*) till 4 (*Helt av samma åsikt*).

|  | Helt av annan åsikt |  |  | Helt av samma åsikt |
| --- | --- | --- | --- | --- |
| Jag lämnar besluten som gäller min hälsa i läkarnas händer | 1 | 2 | 3 | 4 |
| Jag känner mig hörd då jag besöker läkare | 1 | 2 | 3 | 4 |
| Jag är nöjd med den vård jag får av läkare | 1 | 2 | 3 | 4 |
| Jag litar på läkarnas förmåga att ställa korrekta diagnoser | 1 | 2 | 3 | 4 |
| När läkare fattar medicinska beslut har de patientens bästa i åtanke | 1 | 2 | 3 | 4 |
| Läkarna är alldeles för auktoritära mot sina patienter | 1 | 2 | 3 | 4 |

Andra behandlingsformer

Kryssa för de behandlingsformer du under de senaste 12 månaderna har använt dig av i syfte att upprätthålla en god hälsa eller behandla en sjukdom:

□ Vitaminer och mineraler (t.ex. A, B, C, D, E, fluor, jod, zink, kalium, kalcium, magnesium, natrium)
□ Probiotica (t.ex. mjölksyrebakterier)
□ Fiskoljor och fettsyror (t.ex. omega)
□ Kolloidalt silver
□ Gurkmeja
□ Ingefärapreparat
□ Hälsopulver (t.ex. maca, matcha och chlorella)
□ Naturpreparat mot förkylning

□ Aloe vera
□ Kombucha
□ Rå kost (eng. rawfood)

□ Vegansk eller vegetarisk diet
□ Glutenfri diet
□ Laktosfri diet
□ LCHF-diet (låg kolhydrat, fettrik kost)
□ 5:2 diet
□ Medelhavsdiet

□ Paleodiet

□ Fastande
□ Mindfulness
□ Meditation
□ Yoga
□ Tai chi
□ Kiropraktik
□ Akupunktur

□ Koppning
□ Healing (t.ex. fjärrhealing, färghealing, ljudhealing, kristallhealing)

□ Bön

□ Handpåläggning
□ Reiki

□ Rosenmetoden
□ Zonterapi
□ Saltterapi

□ Chakraterapi

□ Homeopati
□ Kinesisk medicin
□ Oil-pulling

□ Ayurveda
□ Detox

Vaccin

Termen *”barnvaccin”* hänvisar till de vaccin som ingår i det nationella vaccinationsprogrammet i Finland för barn upp till 6-års ålder. Detta vaccinationsprogram består av rotavirusvaccinet, pneumokockkonjugatvaccinet (PCV; mot meningit, lunginflammation, blodförgiftning och öroninflammation), DTaP-IPV-Hib vaccinet (”Fem-i-ett-vaccinet”; mot difteri, stelkramp, kikhosta, polio och Hib-sjukdomar som meningit, struplocksinflammation

och blodförgiftning), MPR-vaccinet (mot mässling, påssjuka och röda hund), DtaP-IPV vaccinet (”Fyra-i-ett-vaccinet”; mot difteri, stelkramp, kikhosta och polio) och vattkoppsvaccinet.

Termen *”influensavaccin”* hänvisar till säsongsvaccinen mot influensa.

Vänligen läs påståendena nedan och ange för varje påstående hur mycket du instämmer, genom att ringa in en siffra på skalan 1 (*Helt av annan åsikt*) till 4 (*Helt av samma åsikt*).

|  | Helt av annan åsikt |  |  | Helt av samma åsikt |
| --- | --- | --- | --- | --- |
| Att vaccinera friska barn skyddar andra eftersom det stoppar spridningen av sjukdomen | 1 | 2 | 3 | 4 |
| Mässlingen är en mycket allvarlig sjukdom | 1 | 2 | 3 | 4 |
| Det är bättre att få immunitet via själva sjukdomen än via vaccinet | 1 | 2 | 3 | 4 |
| Det är inte värt att ta influensavaccinet eftersom influensasymtomen är ofarliga | 1 | 2 | 3 | 4 |
| Vaccin kan förorsaka autism | 1 | 2 | 3 | 4 |
| En god hygien gör att mässlingen försvinner ur samhället – att vaccinera sig är inte nödvändigt | 1 | 2 | 3 | 4 |
| Barnvaccinen är trygga att ta | 1 | 2 | 3 | 4 |
| Influensavaccinen är trygga att ta | 1 | 2 | 3 | 4 |
| Risken för bieffekter väger mer än nyttan av barnvaccinen | 1 | 2 | 3 | 4 |
| Risken för bieffekter väger mer än nyttan av influensavaccinen | 1 | 2 | 3 | 4 |
| Vaccin innehåller skadliga mängder kvicksilver | 1 | 2 | 3 | 4 |
| Barn behöver vaccineras mot sjukdomar som inte längre är vanliga | 1 | 2 | 3 | 4 |
| En god handhygien och andra förebyggande åtgärder räcker till för att undvika influensan också utan vaccinering | 1 | 2 | 3 | 4 |
| Barnvaccinen ger ett effektiv skydd mot sjukdomar | 1 | 2 | 3 | 4 |
| Influensavaccinen ger ett effektivt skydd mot sjukdomen | 1 | 2 | 3 | 4 |

Har du någonsin tvekat att låta ditt/dina barn få något av barnvaccinen?

1. Nej

2. Ja

Har du någonsin skjutit upp beslutet att låta ditt/dina barn få något av barnvaccinen?

1. Nej

2. Ja

Har du någonsin nekat till att låta ditt barn få något av barnvaccinen?

1. Nej

2. Ja

Tog du det senaste influensavaccinet?

1. Nej

2. Ja
