# Supplementary material for: Trait reactance and trust in doctors as predictors of vaccination behavior, vaccine attitudes, and use of complementary and alternative medicine in parents of young children
Source: PLoS One. 2020 Jul 27;15(7):e0236527. doi: 10.1371/journal.pone.0236527 (PMC7384640; doi:10.1371/journal.pone.0236527)
Supplement: S3 Questionnaire — (DOCX) [file pone.0236527.s008.docx]

**S3 Questionnaire: Questionnaire in Finnish**

Lääkärit

Lue alla olevat väittämät ja merkitse jokaisen väittämän kohdalle, missä määrin olet samaa tai eri mieltä siitä ympyröimällä numero asteikolla 1:stä (*Täysin eri mieltä*) 4:ään (*Täysin samaa mieltä*).

|  | Täysin eri mieltä |  |  | Täysin samaa mieltä |
| --- | --- | --- | --- | --- |
| Jätän terveyttäni koskevat päätökset lääkäreiden tehtäväksi. | 1 | 2 | 3 | 4 |
| Koen tulevani kuulluksi käydessäni lääkärissä. | 1 | 2 | 3 | 4 |
| Olen tyytyväinen lääkäriltä saamaani hoitoon. | 1 | 2 | 3 | 4 |
| Luotan lääkäreiden kykyyn tehdä oikeita diagnooseja. | 1 | 2 | 3 | 4 |
| Lääkäreiden tehdessä lääketieteellisiä päätöksiä he ajattelevat potilaan parasta. | 1 | 2 | 3 | 4 |
| Lääkärit ovat aivan liian autoritaarisia potilaitaan kohtaan. | 1 | 2 | 3 | 4 |

Muut hoitomuodot

Merkitse rastilla ne hoitomuodot, joita olet käyttänyt viimeisten 12 kuukauden aikana ylläpitääksesi hyvää terveyttä tai hoitaaksesi sairautta:

□ Vitamiinit ja mineraalit (esim. A, B, C, D, E, fluori, jodi, sinkki, kalium, kalsium, magnesium, natrium)
□ Probiootit (esim. maitohappobakteerit)
□ Kalaöljyt ja rasvahapot (esim. omega)
□ Kolloidinen hopea
□ Kurkuma
□ Inkiväärivalmiste
□ Terveysjauheet (esim. maca, matcha ja klorella)
□ Luonnonvalmiste flunssan ehkäisemiseksi

□ Aloe vera
□ Kombutsa
□ Raakaravinto (engl. rawfood)

□ Vegaani- tai kasvisruokavalio
□ Gluteeniton ruokavalio
□ Laktoositon ruokavalio
□ LCHF-ruokavalio (vähän hiilihydraatteja, paljon rasvaa)
□ 5:2-ruokavalio
□ Välimeren ruokavalio

□ Paleoruokavalio

□ Paasto
□ Mindfulness
□ Meditaatio
□ Jooga
□ Tai chi
□ Kiropraktiikka
□ Akupunktio

□ Kuppaus
□ Healing (esim. kaukohoito, värihoito, äänihoito, kristallihoito)

□ Rukous

□ Energiahoito
□ Reiki

□ Rosen-menetelmä
□ Vyöhyketerapia
□ Suolaterapia

□ Chakra-terapia

□ Homeopatia
□ Kiinalainen lääketiede
□ Öljypurskuttelu (oil-pulling)

□ Ayurveda
□ Detox

Rokotteet

Termillä *”lasten rokotteet”* viitataan niihin rokotteisiin, jotka sisältyvät Suomessa toteutettavaan, enintään 6-vuotiaille lapsille tarkoitettuun kansalliseen rokotusohjelmaan. Ohjelma koostuu rotavirusrokotteesta, pneumokokkikonjugaattirokotteesta (PCV; aivokalvontulehdusta, keuhkokuumetta, verenmyrkytystä ja korvatulehdusta vastaan), DTaP-IPV-Hib-rokotteesta (”viitosrokote”; kurkkumätää, jäykkäkouristusta, hinkuyskää, poliota ja Hib-tauteja, kuten aivokalvontulehdusta, kurkunkannentulehdusta ja verenmyrkytystä vastaan), MPR-rokotteesta (tuhkarokkoa, sikotautia ja vihurirokkoa vastaan), DtaP-IPV-rokotteesta (”nelosrokote”; kurkkumätää, jäykkäkouristusta, hinkuyskää ja poliota vastaan), sekä vesirokkorokotteesta.

Termillä *”influenssarokotteet”* viitataan influenssaa vastaan annettaviin kausirokotteisiin.

Lue alla olevat väittämät ja merkitse jokaisen väittämän kohdalle, missä määrin olet samaa tai eri mieltä siitä ympyröimällä numero asteikolla 1:stä (*Täysin eri mieltä*) 4:ään (*Täysin samaa mieltä*).

|  | Täysin eri mieltä | Osittain eri mieltä | Osittain samaa mieltä | Täysin samaa mieltä |
| --- | --- | --- | --- | --- |
| Terveiden lasten rokottaminen suojaa muita, koska se pysäyttää tautien leviämisen. | 1 | 2 | 3 | 4 |
| Tuhkarokko on erittäin vaarallinen tauti. | 1 | 2 | 3 | 4 |
| On parempi saada immuniteetti itse tauti sairastamalla kuin rokotteen kautta. | 1 | 2 | 3 | 4 |
| Influenssarokotetta ei kannata ottaa, koska influenssan oireet ovat vaarattomia. | 1 | 2 | 3 | 4 |
| Rokote voi aiheuttaa autismia. | 1 | 2 | 3 | 4 |
| Tuhkarokko häviää yhteiskunnasta hyvän hygienian avulla – itsensä rokotuttaminen ei ole tarpeen. | 1 | 2 | 3 | 4 |
| Lasten rokotteet ovat turvallisia. | 1 | 2 | 3 | 4 |
| Influenssarokotteet ovat turvallisia. | 1 | 2 | 3 | 4 |
| Sivuvaikutusten riski painaa enemmän kuin lasten rokotteiden hyöty. | 1 | 2 | 3 | 4 |
| Sivuvaikutusten riski painaa enemmän kuin influenssarokotteiden hyöty. | 1 | 2 | 3 | 4 |
| Rokotteet sisältävät haitallisia määriä elohopeaa. | 1 | 2 | 3 | 4 |
| Lapset täytyy rokottaa sellaisia tauteja vastaan, jotka eivät ole enää tavallisia. | 1 | 2 | 3 | 4 |
| Hyvä käsihygienia ja muut ennalta ehkäisevät toimet riittävät suojautumaan influenssalta myös ilman rokotusta. | 1 | 2 | 3 | 4 |
| Lasten rokotteet suojaavat tehokkaasti taudeilta. | 1 | 2 | 3 | 4 |
| Influenssarokotteet suojaavat tehokkaasti taudilta. | 1 | 2 | 3 | 4 |

Oletko koskaan epäröinyt ottaa lapsellesi jotakin lasten rokotetta?

1. En

2. Kyllä

Oletko koskaan lykännyt päätöstä ottaa lapsellesi jotakin lasten rokotetta?

1. En

2. Kyllä

Oletko koskaan kieltäytynyt ottamasta lapsellesi jotakin lasten rokotetta?

1. En

2. Kyllä

Otitko viimeisimmän influenssarokotteen?

1. En

2. Kyllä
