# Supplementary material for: Trait reactance and trust in doctors as predictors of vaccination behavior, vaccine attitudes, and use of complementary and alternative medicine in parents of young children
Source: PLoS One. 2020 Jul 27;15(7):e0236527. doi: 10.1371/journal.pone.0236527 (PMC7384640; doi:10.1371/journal.pone.0236527)
Supplement: Attachment — Submitted filename: Response to Reviewers.docx [file pone.0236527.s010.docx]

PONE-D-20-10006R1
Trait reactance and trust in doctors as predictors of vaccination behavior, vaccine attitudes, and use of complementary and alternative medicine in parents of young children
PLOS ONE

Dear Dr. Soveri,

Thank you for submitting your manuscript to PLOS ONE. After careful consideration, we feel that it has merit but does not fully meet PLOS ONE’s publication criteria as it currently stands. Therefore, we invite you to submit a revised version of the manuscript that addresses the points raised during the review process.

Please submit your revised manuscript by Aug 18 2020 11:59PM. If you will need more time than this to complete your revisions, please reply to this message or contact the journal office at plosone@plos.org. When you're ready to submit your revision, log on to https://www.editorialmanager.com/pone/ and select the 'Submissions Needing Revision' folder to locate your manuscript file.

Please include the following items when submitting your revised manuscript:

- A rebuttal letter that responds to each point raised by the academic editor and reviewer(s). You should upload this letter as a separate file labeled 'Response to Reviewers'.
- A marked-up copy of your manuscript that highlights changes made to the original version. You should upload this as a separate file labeled 'Revised Manuscript with Track Changes'.
- An unmarked version of your revised paper without tracked changes. You should upload this as a separate file labeled 'Manuscript'.

If you would like to make changes to your financial disclosure, please include your updated statement in your cover letter. Guidelines for resubmitting your figure files are available below the reviewer comments at the end of this letter.

If applicable, we recommend that you deposit your laboratory protocols in protocols.io to enhance the reproducibility of your results. Protocols.io assigns your protocol its own identifier (DOI) so that it can be cited independently in the future. For instructions see: <http://journals.plos.org/plosone/s/submission-guidelines#loc-laboratory-protocols>

We look forward to receiving your revised manuscript.

Kind regards,

Peter Karl Jonason
Academic Editor
PLOS ONE


[Note: HTML markup is below. Please do not edit.]

Reviewers' comments:

Reviewer's Responses to Questions

**Comments to the Author**

1. If the authors have adequately addressed your comments raised in a previous round of review and you feel that this manuscript is now acceptable for publication, you may indicate that here to bypass the “Comments to the Author” section, enter your conflict of interest statement in the “Confidential to Editor” section, and submit your "Accept" recommendation.

Reviewer #1: All comments have been addressed

Reviewer #2: All comments have been addressed

2. Is the manuscript technically sound, and do the data support the conclusions?

The manuscript must describe a technically sound piece of scientific research with data that supports the conclusions. Experiments must have been conducted rigorously, with appropriate controls, replication, and sample sizes. The conclusions must be drawn appropriately based on the data presented.

Reviewer #1: Yes

Reviewer #2: Yes

3. Has the statistical analysis been performed appropriately and rigorously?

Reviewer #1: Yes

Reviewer #2: Yes

4. Have the authors made all data underlying the findings in their manuscript fully available?

The [PLOS Data policy](http://www.plosone.org/static/policies.action#sharing) requires authors to make all data underlying the findings described in their manuscript fully available without restriction, with rare exception (please refer to the Data Availability Statement in the manuscript PDF file). The data should be provided as part of the manuscript or its supporting information, or deposited to a public repository. For example, in addition to summary statistics, the data points behind means, medians and variance measures should be available. If there are restrictions on publicly sharing data—e.g. participant privacy or use of data from a third party—those must be specified.

Reviewer #1: Yes

Reviewer #2: Yes

5. Is the manuscript presented in an intelligible fashion and written in standard English?

PLOS ONE does not copyedit accepted manuscripts, so the language in submitted articles must be clear, correct, and unambiguous. Any typographical or grammatical errors should be corrected at revision, so please note any specific errors here.

Reviewer #1: Yes

Reviewer #2: Yes

6. Review Comments to the Author

Please use the space provided to explain your answers to the questions above. You may also include additional comments for the author, including concerns about dual publication, research ethics, or publication ethics. (Please upload your review as an attachment if it exceeds 20,000 characters)

Reviewer #1: The authors have taken care in the revision and have addressed all my original concerns. My only remaining comment: in revising the abstract, the conclusions section contains quite a bit of causal language, which should be avoided given that it's a correlational study. Apart from this I have no further comments and I don't need to see a revision. I look forward to seeing the paper in print at some stage soon.
All the best, Matthew

*Response: We have now revised the Conclusions section in the Abstract so that there is no causal language in the sentences that relate to our results. We decided to keep the causal language in the last sentence, because that sentence is based on speculation about the meaning of the results. The text now reads as follows:*

*”Taken together, higher trait reactance seems to be relevant for attitudes and behaviors that go against conventional medicine, because trait reactance is connected to a distrust in medical doctors. Our findings also suggest that high trait reactance and low trust in doctors function differently for different people: For some individuals they might be associated with anti-vaccination attitudes and behavior, while for others they might be related to CAM use. We speculate that this is because people differ in what is important to them, leading them to react against different aspects of conventional medicine.”*

Reviewer #2: Thanks for addressing my comments seriously. As well as being more polished overall, the revised analyses and results provide a much clearer and less ambiguous interpretation, which well supports the conclusions. An interesting result, and good work.

7. PLOS authors have the option to publish the peer review history of their article ([what does this mean?](https://journals.plos.org/plosone/s/editorial-and-peer-review-process#loc-peer-review-history)). If published, this will include your full peer review and any attached files.


If you choose “no”, your identity will remain anonymous but your review may still be made public.


**Do you want your identity to be public for this peer review?** For information about this choice, including consent withdrawal, please see our [Privacy Policy](https://www.plos.org/privacy-policy).

Reviewer #1: **Yes:** Matthew Hornsey

Reviewer #2: No
